# Synchronized roles of pannexin and connexin in nasal mucosal epithelia

**DOI:** 10.1007/s00405-018-4947-y

**Published:** 2018-03-24

**Authors:** Toyoaki Ohbuchi, Hideaki Suzuki

**Affiliations:** Department of Otorhinolaryngology-Head and Neck Surgery, School of Medicine, University of Occupational and Environmental Health, 1-1 Iseigaoka, Yahatanishi-ku, Kitakyushu, 807-8555 Japan

**Keywords:** ATP release, Ciliary beat frequency, Connexin, Gap junction, Mucociliary clearance, Mucus blanket, Nasal mucosal epithelia, Pannexin-1

## Abstract

**Background:**

Nasal mucosal epithelial cells express connexins, the prototypical gap junction proteins, and pannexins, a new family of channel proteins homologous to the invertebrate gap junction proteins. The physiological and pathophysiological roles of these transmembrane proteins in nasal mucosa are largely still unknown.

**Purpose:**

Pannexins participate in ATP release into the extracellular space in various tissues, and ATP plays important roles in mucociliary clearance, especially by regulating ciliary beat activity. Therefore, we focused on the functional relationship between connexins, pannexin-1, ATP release, and mucociliary clearance in nasal epithelia.

**Results and Conclusions:**

Connexins participate in the generation of intercellular calcium waves, in which calcium-mediated signaling responses spread to contiguous cells through the gap junction formed by connexins to transmit calcium signaling throughout the airway epithelium. Pannexins in the nasal mucosa may contribute to not only ciliary beat modulation via ATP release, but also regulation of mucus blanket components via H_2_O efflux. The synchronized roles of pannexin and connexin may provide a new insight into effective mucociliary clearance systems in nasal mucosa.

In the upper airway, mucociliary clearance (MCC) serves to remove inhaled particulate matter along with secreted mucus, and thereby contributes to host defense mechanisms. MCC is regulated by appropriate levels of mucus production and ciliary activity, which is usually measured by ciliary beat frequency (CBF). In the nose, mucociliary dysfunction can be a significant clinical problem, and has been reported to occur in patients with various inflammatory diseases including chronic rhinosinusitis (CRS) [1].

The connexin proteins form hemichannels that dock to form gap junctions, which are intercellular communication channels [2]. These junctions mediate electrical and biochemical communication between a wide variety of somatic cells and tissues [2]. There are 21 connexin subtypes that have been identified in humans. A recent report revealed that 16 connexin genes are expressed in the human sinus mucosa [3]. One report found that connexin-43 is significantly upregulated in CRS patients compared to healthy controls at both the mRNA and protein levels, whereas other reports searching for a potential cause of dysfunction in the sinonasal epithelium failed to find a significant difference in the expressions of connexin-26, -30, -32, or -43 [3–5]. Although the relationship between CRS and connexin expression is still controversial, those reports have consistently shown that connexin-43 is expressed in human nasal epithelial cells. Connexin-43 is also expressed in rat nasal epithelia [6].

An intracellular response to calcium influx in nasal mucosal cells induces an increase in CBF and triggers a calcium wave that spreads to neighboring cells through the gap junction channels, such as those containing connexin-43. This wave elicits a calcium influx reaction in these neighboring cells via the same mechanism, leading to the transmission of calcium signaling throughout the airway epithelium [7, 8]. Many chemical stimuli can initiate this calcium signal. For example, extracellular ATP is a key modulator of CBF through the activation of purinergic receptors, which leads to calcium influx in the epithelial cell [1, 9, 10].

ATP is thought to be released into the extracellular space via two distinct pathways: vesicular- and channel-mediated release pathways. Among the many channel-mediated pathways, the pannexin channel is considered a promising candidate mediator of ATP release. Pannexins are a family of transmembrane channel proteins in vertebrates comprising three subtypes: pannexin-1, pannexin-2, and pannexin-3 [11]. Pannexins are homologous to innexins, the invertebrate gap junction proteins, and thus were originally cloned as gap junction-related proteins; however, they do not appear to play a role as gap junction proteins in vivo so far [11–14]. Consistent with these findings, the pannexins have no significant sequence similarity to connexins [15].

The pannexin-1 channel forms a homohexameric large-conductance nonselective channel that participates in ATP release into the extracellular space [12, 16–18]. Recently, we have provided evidence of pannexin-1 expression in, and ATP release from, rat and human nasal mucosa [19–22]. The pannexin-1 channel can be activated in either a calcium-dependent or, interestingly, calcium-independent manner. In calcium-dependent activation, pannexin-1 channel opening is evoked by signal transduction events following the activation of the ionotropic purinergic P2 x 7 receptor, which is directly coupled with the pannexin-1 channel [12, 16, 23]. Activation of the acetylcholine receptor and transient receptor potential vanilloid 1 (TRPV1) also induces ATP release by pannexin-1 in nasal mucosa [21, 22]. In calcium-independent activation, the pannexin-1 channel is triggered by mechanical stimulation, such as hypotonic stress-induced cell swelling and membrane stretching [2, 13, 20, 24–26].

The role of pannexin-dependent ATP release in the nasal mucosa is still a matter of debate. The mucus blanket is divided into a gel-like outer mucus layer (OML) and a liquid periciliary fluid layer (PCFL) [27]. The mucus of the OML is produced and secreted by goblet cells and submucosal glands. The fluid of the PCFL is maintained by H_2_O balance as well as an equilibrium between anion efflux and cation influx through the epithelial cell membrane. If the volume of the PCFL is reduced, ciliary movement becomes inefficient because of increased contact between the cilia and the viscous OML. Conversely, when the volume of the PCFL increases beyond its normal volume, ciliary movement also becomes inefficient because the cilia cannot reach the OML to stimulate it. Therefore, PCFL volume is extremely important for ensuring functional MCC.

These facts led us to hypothesize that pannexin-dependent ATP release may, in part, constitute a H_2_O homeostasis system for maintaining the appropriate PCFL volume. To address this hypothesis, we examined whether pannexin-1 channels on nasal columnar epithelial cells contribute to H_2_O efflux. Our recent studies showed that TRPV1-associated, capsaicin-induced ATP release thorough pannexin-1 regulates ciliary beat activity in rat nasal mucosa [22]. Therefore, we used individual rat columnar cells in this study, which were enzymatically dissociated from the nasal mucosa. We used 10 µM capsaicin as a TRPV1 agonist, 100 µM ruthenium red as a TRPV1 antagonist, and 10 µM carbenoxolone (CBX) as a pannexin-1 channel antagonist. We expect that connexin hemichannels, likely those containing connexin-43, are expressed in the single nasal epithelial cells. However, 10 µM CBX is considered insufficient to block connexin hemichannels [28, 29].

Application of capsaicin led to shrinking of the columnar epithelial cells. Interestingly, this shrinking occurred only at the apical side but not the basal side. This shrinking was inhibited by ruthenium red or CBX when they were added in combination with the capsaicin (Fig. 1). We could not completely rule out the involvement of connexin hemichannels because the blockers used in our protocol do not exhibit strict pharmacological specificity. However, if connexin hemichannels were involved in this phenomenon, cell shrinkage would be expected to occur throughout the cell membrane rather than only at the apical side.

**Fig. 1:**
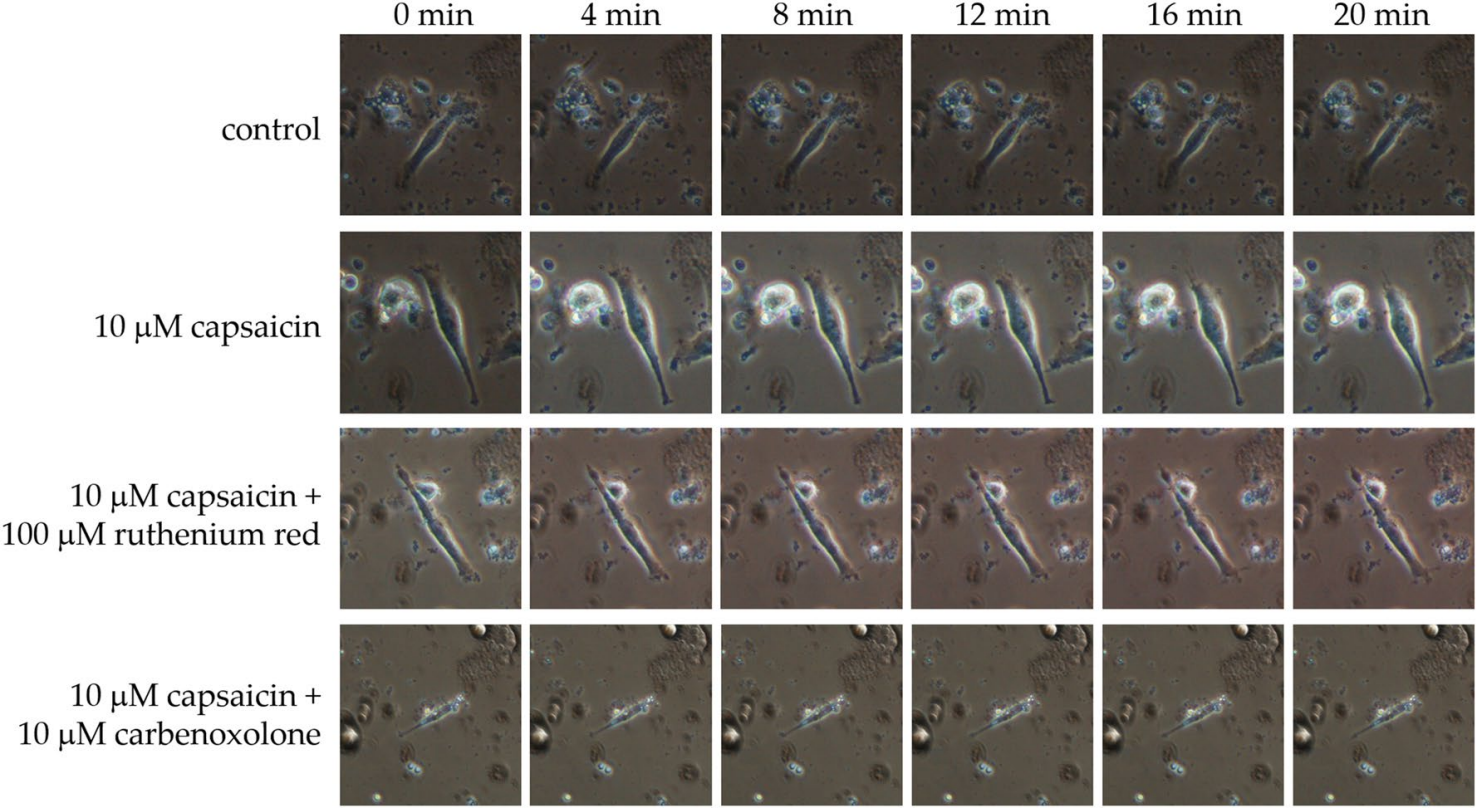
Representative dissociated rat nasal columnar epithelial cells under a phase-contrast microscope with the indicated treatments. Photographs were captured every 4 min from 0 min (baseline) to 20 min. The cell shape did not change in the control cells (*n* = 2), and application of 10 µM capsaicin led the apical side of the cells to shrink over the observation period (*n* = 3). This capsaicin-induced local shrinkage was inhibited by co-treatment with either 100 µM ruthenium red (*n* = 2) or 10 µM carbenoxolone (*n* = 2)

Although we were not able to definitively determine the role of pannexin-1, these results are consistent with our hypothesis described above. One possible explanation for these results is that the pannexin-1 channel is involved in release of the anionic form of ATP, ATP^−^, together with H_2_O efflux to the extracellular space on the apical side of the cell under low-PCFL conditions, because the apical side is more exposed to mechanical stress on the nasal mucosa (Fig. 2).

**Fig. 2:**
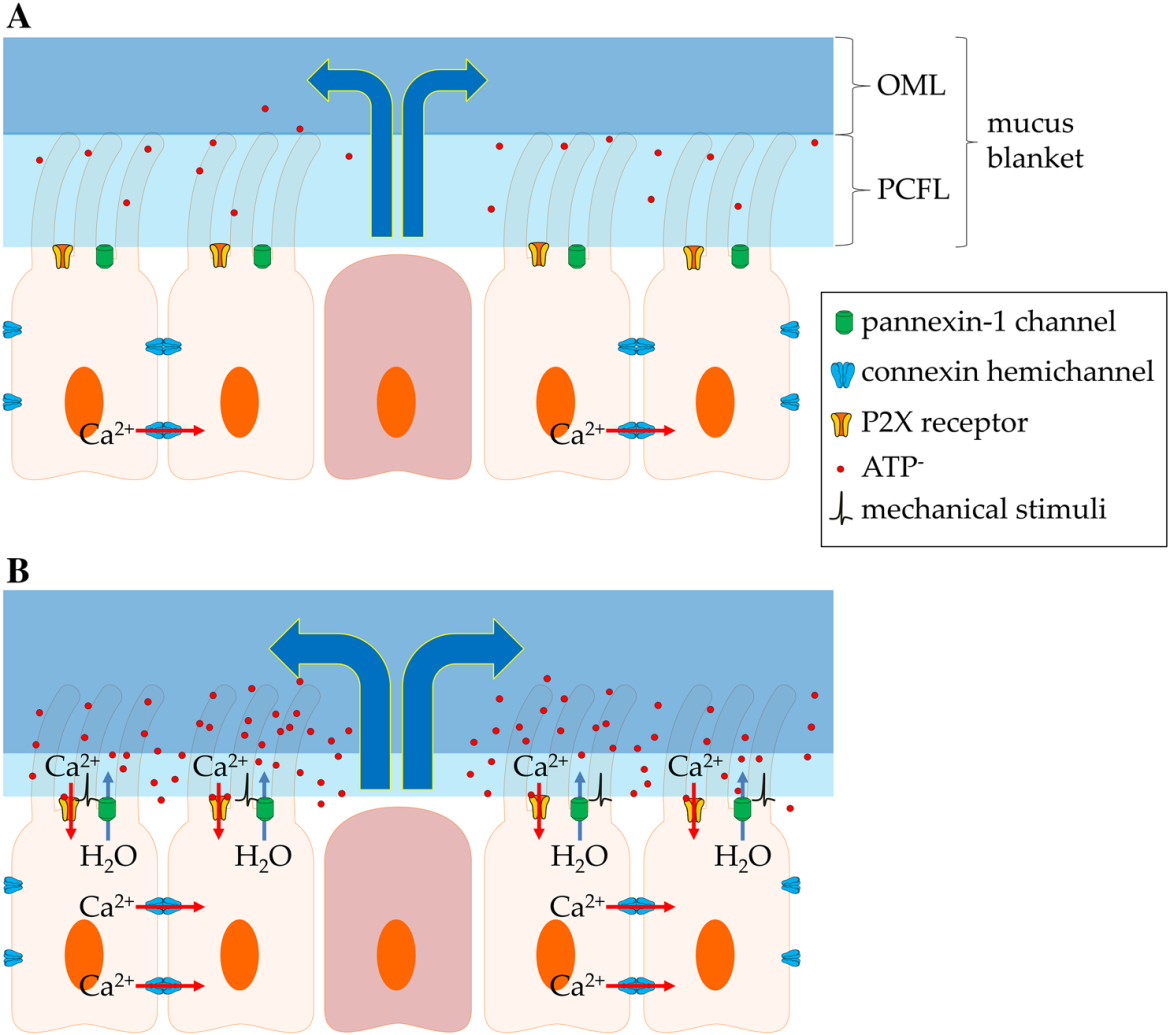
Schematic of our hypothesized relationship between the mucus blanket components and ATP^−^/H_2_O efflux. **a** Under normal conditions, the gel-like outer mucus is appropriately secreted by goblet cells (the central cell in the figure) and submucosal glands. Liquid periciliary fluid is also maintained at moderate levels. Together, these allow efficient removal of inhaled particulate matter along with secreted mucus. **b** In pathological conditions such as infection and inflammation, the outer mucus is produced and secreted in excess followed by a decrease of periciliary fluid. The changes in osmolality and viscosity cause cell membrane stretching, leading to mechanical stimulation that induces pannexin-1 opening. Anionic ATP^−^ would then be released via the pannexin-1 channel into the periciliary fluid layer, along with H_2_O that would increase the volume of the periciliary fluid. The released ATP could activate purinergic receptors, and ciliary movement would improve

In conclusion, we believe that pannexin-1 channel-mediated ATP release has a distinct role in MCC from that of connexin channels and hemichannels. Synergetic effects of the pannexin-1 channel and the connexin channel could be the key molecular factors in the regulatory mechanism of the upper airway function. Modulation of these molecules may open a novel therapeutic strategy in the management of upper respiratory disorders.

## Abbreviations

MCC: Mucociliary clearance
CBF: Ciliary beat frequency
CRS: Chronic rhinosinusitis
CBX: Carbenoxolone
OML: Outer mucus layer
PCFL: Periciliary fluid layer
